# Assessment of stone ablation rate using the Moses technology modes with different energy and pulse settings: An experimental study

**DOI:** 10.1080/20905998.2023.2301641

**Published:** 2024-01-05

**Authors:** BegonaBallesta Martinez, Panteleimon Ntasiotis, Paraskevi Katsakiori, Vasileios Tatanis, Angelis Peteinaris, Solon Faitatziadis, Kristiana Gkeka, Theodoros Spinos, Theofanis Vrettos, Evangelos Liatsikos, Panagiotis Kallidonis

**Affiliations:** a Department of Urology, University of Patras, Patras, Spain; b Department of Urology, University of Patras, Patras, Greece; c Department of Anesthesiology and ICU, University of Patras, Patras, Greece; d Department of Urology, Medical University of Vienna, Vienna, Austria

**Keywords:** Laser lithotripsy, pulse modulation, Moses technology

## Abstract

**Objectives:**

To compare lithotripsy ablation rate with the Moses modes versus conventional pulse modes when using the Holmium:Yttrium–Aluminum–Garnet (Ho:YAG) laser.

**Methods:**

The Lumenis® Pulse P120H Holmium Laser System and a 365 μm Moses D/F/L fiber were used to assess stone ablation rate in conventional Short and Long Pulse as well as Moses Contact and Distance at 10 W (0.5Jx20Hz and 2Jx5Hz) and 60 W (1Jx60Hz and 2Jx30Hz). Hard and soft phantom stones were formed, and all tests were conducted in a custom experimental configuration installed in a saline-filled bath. The laser was delivered up to 3 kJ of total energy. The fragmentation pattern was assessed via photographs in each cohort.

**Results:**

The time to reach the target energy was 5 min and 50 s in all 10 W and 60 W trials, respectively. In both stone types, ablation was more effective when high-power, high-energy and Moses Distance was utilized. In soft stones, the lowest ablation rate was detected in the Long Pulse modality in all power, energy and frequency settings. Overall, when dusting settings (high-frequency, low-energy) were used, a deeper single cavitation was observed rather than small cavitations.

**Conclusions:**

The most effective pulse modality as evaluated via stone ablation rate depends on the stone hardness as well as energy and frequency settings. In both hard and soft stones, ablation is more effective when 60 W (2Jx30Hz) power settings and Moses Distance are used. Tailored laser settings in terms of energy and frequency could be set for each case scenario.

## Introduction

Endourology is currently the preferred treatment strategy for most mid- to large-sized renal stones and ureteral calculi [1,2]. The integration of novel miniaturized instruments and new high-power (HP) lasers has revolutionized the field of lithotripsy, leading to significant decrease in operative time as well as improvement of operative outcomes [3]. Currently, the Holmium:Yttrium–Aluminum–Garnet (Ho:YAG) laser is widely utilized as it is characterized by HP settings [4]. Ho:YAG laser emits radiation at a wavelength of 2120 nm. Besides, it is strongly absorbed by water and it is associated with a depth penetration of 0.4 mm. It can achieve both short- and long-pulse emissions, while its total power varies from 20 to 150 W [5]. The mechanism of action of Ho:YAG is based on two parallel axes, i.e. the photothermal detachment and the thermomechanical delamination. According to the former, the application of Ho:YAG increases the temperature of both the surrounding water and the stone itself, thus, leading to stone chemical decomposition. Based on the latter, the water inside the pores, cracks and layers of the stone’s surface absorbs the laser energy causing micro-explosions [6].

Despite being a powerful energy source, the special lithotripsy conditions may restrict the efficacy of laser devices. The main factors that limit the efficacy of Ho:YAG laser are the energy transmission through water which is affected by the distance between the fiber and the stone [3,7,8] and the stone retropulsion which parallelly leads to increased fiber-stone distance. To overcome these limitations, devices with new integrated functions have been designed and presented to the market [1]. One of the most important technological advancements has been the Moses Technology^Ⓡ^. Based on the ‘Moses effect’ principle [9], it generates a controlled pulse-energy release and transfer in two consecutive phases [1]. Thus, the energy is not delivered to the stone by the conventional uni-phase pulse mode but in a dual-phase one. The system results in the improvement of energy delivery to the stone as well as stone retropulsion reduction leading to an enhanced efficiency of laser ablation [1,3,7,8,10–12]. Nevertheless, there are important questions regarding Moses Technology^Ⓡ^ that remain either controversial or unanswered. The indications and advantages of Moses pulse, its efficacy in all types and sizes of stones, and the appropriate settings based on the stone hardness are points to be clarified.

To provide the urology community with more data on the way Moses Technology^Ⓡ^ affects the efficiency of stone lithotripsy, the objective of our study was to compare the lithotripsy ablation rates of the Moses Technology^Ⓡ^ in Contact and Distance Modes to those of conventional short- and long-pulse modes of holmium laser in soft and hard stones using HP and low-power (LP) lithotripsy settings.

## Materials and methods

### Stones

An in vitro study was conducted utilizing Lumenis^Ⓡ^ Pulse P120H holmium laser system (Lumenis Ltd, Yokneam, Israel). A 365 μm Moses D/F/L fiber (Lumenis Ltd, Yokneam, Israel) was employed. Hard and soft phantom stones with the same shape and size were constructed out of Lumenis® powder (BEGO Lincoln, RI, USA). The composition of the stones was designed as described in the literature [13–16]. Hard stones were prepared with 15 g of powder and 3 ml water mixed up in an automat shaker, whereas soft phantom stones were composed of 15 g of powder and 6 ml water using the same shaking method. After drying for 72 h, the artificial stones were hydrated for 1 h right after the beginning of the experiment.

### Experimental set-up

**Figure 1.**
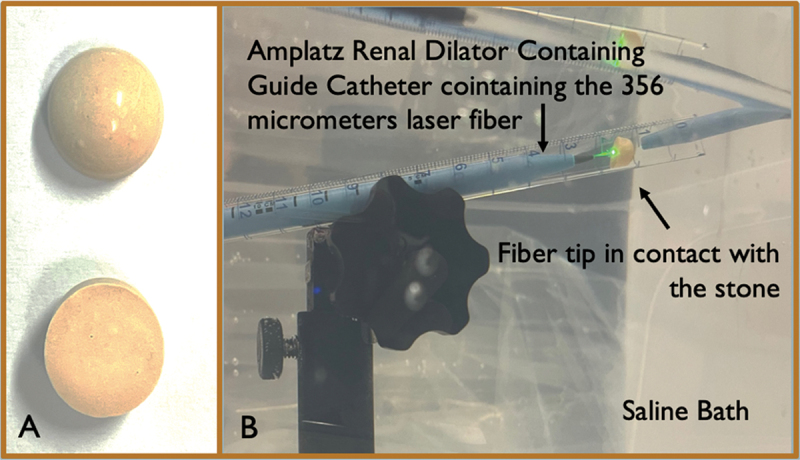
Experimental set-up (A. Phantom BegoStones, B. Custom experimental configuration installed in a saline-filled bath where all the tests were conducted).

The experimental set-up is presented in Figure 1. The experiment was performed inside a transparent container filled with normal saline. The stones were inserted into a 10:01 crystal tube. The laser fiber was placed into an 8 Fr ureteral catheter (Cook Medical, Indiana, USA) and then, inserted through an 18 Fr Amplatz Renal Dilator (Cook Medical, Indiana, USA) in the crystal tube. An electronic scale was used to weigh each stone in dry conditions prior to and post the experiment.

### Performance of the experiment

Ablation rate (AR) was evaluated in four pulse modality settings, i.e. ‘Short Pulse’, ‘Long Pulse’, ‘Moses Contact’ and ‘Moses Distance’. As recommended by Winship *et al*., the laser fiber tip was positioned in contact with the stone at Moses Contact and at 1 mm distance from the stone at Moses Distance [16]. The evaluated settings were LP of 10 W (0.5Jx20Hz and 2Jx5Hz) and HP of 60 W (1Jx60Hz and 2Jx30Hz). The laser was delivered up to a total energy of 3 kJ in all cases. All fragments and dust were collected and photographed after the lithotripsy. Stone AR (mgr/s) was defined as the stone mass difference before and 72 h after the experiment over lasing time. Measurements were performed blindly as researchers recording the measurements did not know the settings and pulse modality used in each experiment.

### Statistical analysis

Statistical analyses were performed with SPSS v25 software (IBM Statistics, NY, USA). The numerical variables were presented as median (range).

## Results

A total of 96 trials were conducted. The time to reach the target energy of 3 kJ was 5 min and 50 s in all the 10 W and 60 W trials, respectively. Table 1 displays the median stone mass prior to and post-lasering. Table 2 presents the median ablation rate of each group.

**Table 1.** Stone mass (mgr, median (range)) prior to and post-lasering.

		Low-power lithotripsy (10 W)				High-power lithotripsy (60 W)			
		O.5Jx20Hz		2Jx5Hz		1Jx60Hz		2Jx30Hz	
Stone type	Pulse mode	Mass before lasering	Mass after lasering	Mass before lasering	Mass after lasering	Mass before lasering	Mass after lasering	Mass before lasering	Mass after lasering
15:06	Moses Contact	610 (580–620)	560 (540–560)	450 (450–470)	280 (260–280)	660 (640–660)	500 (490–500)	490 (490–500)	330 (330–360)
15:06	Moses Distance	500 (480–520)	470 (440–480)	510 (500–530)	270 (270–320)	490 (460–500)	350 (320–350)	490 (470–520)	240 (190–250)
15:06	Short pulse	480 (460–520)	460 (430–470)	510 (480–550)	360 (320–390)	480 (460–510)	350 (320–380)	430 (430–480)	280 (260–290)
15:06	Long pulse	480 (420–510)	460 (400–460)	490 (450–520)	330 (330–420)	660 (630–710)	570 (490–600)	470 (450–560)	330 (300–380)
15:03	Moses Contact	570 (550–570)	550 (530–560)	670 (640–690)	510 (500–520)	680 (650–700)	600 (530–620)	570 (540–610)	460 (360–460)
15:03	Moses Distance	590 (580–640)	510 (460–540)	590 (580–610)	460 (420–490)	700 (680–750)	610 (570–660)	580 (550–590)	360 (300–400)
15:03	Short pulse	470 (460–500)	440 (400–450)	630 (560–680)	570 (430–580)	570 (560–630)	440 (400–540)	580 (540–610)	480 (400–480)
15:03	Long pulse	590 (580–610)	560 (530–560)	630 (570–660)	410 (350–510)	590 (590–630)	440 (410–450)	530 (490–590)	360 (280–380)

**Table 2.** Ablation rate (mgr/s, median (range)) of the different modes and settings.

		Low-power lithotripsy (10 W)		High-power lithotripsy (60 W)	
Stone type	Pulse mode	0.5Jx20Hz	2Jx5Hz	1Jx60Hz	2Jx30Hz
15:06	Moses Contact	0.17 (0.13–0.2)	0.63 (0.57–0.63)	3.2 (2.8–3.4)	3.2 (2.6–3.4)
15:06	Moses Distance	0.13 (0.07–0.17)	0.77 (0.7–0.8)	2.8 (2.2–3.6)	5.4 (5–5.6)
15:06	Short pulse	0.1 (0.07–0.17)	0.53 (0.4–0.63)	2.6 (2.2–3.2)	3.4 (2.8–4)
15:06	Long pulse	0.07 (0.07–0.17)	0.4 (0.33–0.53)	2.2 (1.8–2.8)	3 (2.8–3.6)
15:03	Moses Contact	0.07 (0.03–0.07)	0.57 (0.4–0.6)	1.6 (1.6–2.4)	3 (2.2–3.6)
15:03	Moses Distance	0.33 (0.23–0.43)	0.5 (0.3–0.57)	1.8 (1.4–2.6)	4.6 (3.6–5)
15:03	Short pulse	0.17 (0.07–0.23)	0.33 (0.20–0.43)	2.4 (1.8–3.4)	2.6 (2–2.8)
15:03	Long pulse	0.17 (0.07–0.2)	0.73 (0.5–0.73)	3.6 (3–3.6)	4.2 (3–4.6)

### HP vs LP lithotripsy

**Figure 2.**
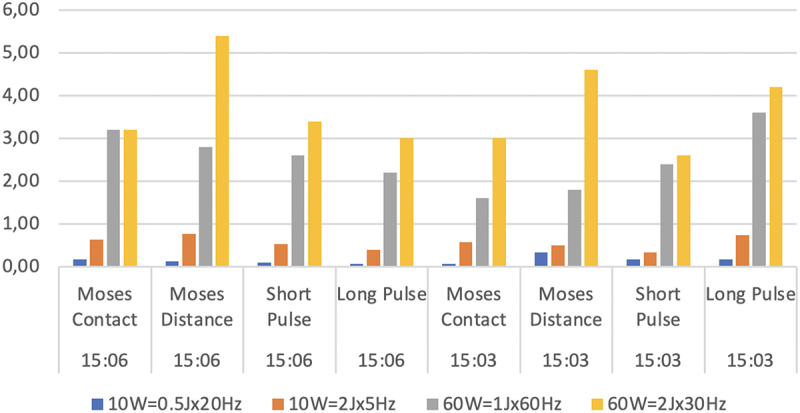
Median ablation rates (mg/s) for hard and soft stones using four pulse modes (10 W = 0.5Jx20Hz, 10 W = 2Jx5Hz, 60 W = 1Jx60Hz and 60 W = 2Jx30Hz).

The total power used for lithotripsy was strongly associated with the ARs independently of the laser mode or stone types. The greatest difference in median AR was detected between 10 W (0.5Jx2Hz) and 60 W (2Jx30Hz) in soft stones with Moses Distance mode (0.13 mg/s vs 5.4 mg/s) whilst the smallest difference was presented in hard stones with short-pulse mode (0.17 mg/s vs 2.6 mg/s) (Figure 2).

### The role of the energy

In both LP and HP lithotripsy, the high-energy settings were associated with higher median AR in most of the groups. In LP lithotripsy, this fact was confirmed in all groups. The greatest difference was detected in soft stones with Moses Distance mode (0.13 mg/s vs 0.77 mg/s), whereas the smallest difference was presented in hard stones with short-pulse mode (0.17 mg/s vs 0.33 mg/s). In HP lithotripsy, a similar pattern was noticed in all trials except one. In the case of soft stones treated with Moses Contact, the median AR was the same irrespective of the energy settings used (3.2 mg/sin both 1Jx60Hz and 2Jx30Hz) (Figure 2).

### Τhe Moses Technology^Ⓡ^

The Moses Technology^Ⓡ^ was noticed as a superior mode compared to the standard pulse modes in soft stones. More precisely, in low-energy settings, the Moses Contact mode was associated with the highest median AR (0.17 mg/s for 10 W and 3.2 mg/s for 60 W). In high-energy settings, the Moses Distance was proved to lead to the highest median AR (0.77 mg/s for 10 W and 5.4 mg/s for 60 W). In contrast, in hard stones, a unique pattern was not detected. The Moses Distance mode was proven to be superior in low-energy LP (0.33 mg/s) and high-energy HP (4.6 mg/s). The long pulse was the superior mode in high-energy LP (0.73 mg/s) and low-energy HP (3.6 mg/s) (Figure 2).

### Fragmentation

**Figure 3.**
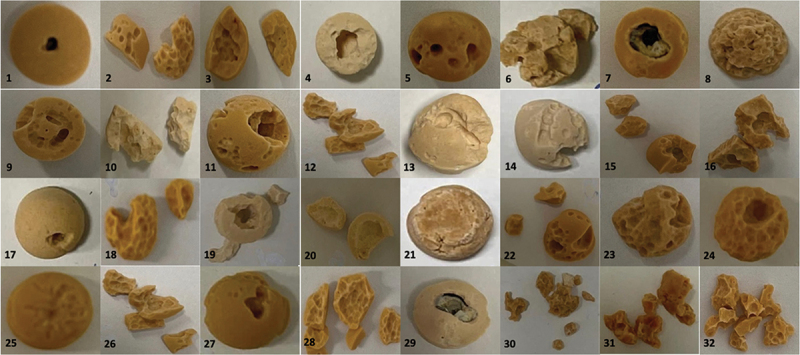
Fragmentation patterns (stone lasered until 3 kJ).

Fragmentation patterns are shown in Figure 3. Overall, when dusting settings (high-frequency, low-energy) were used, a deeper single cavitation was observed rather than small cavitations. The long pulse and Moses distance modalities resulted in more small fragments compared to short pulse and Moses Contact.

Legend:

**Row 1**: Moses Contact: Soft stone (1. 10 W = 0.5Jx20Hz, 2. 10 W = 2Jx5Hz, 3. 60 W = 1Jx60Hz, 4. 60 W = 2Jx30Hz); Hard Stone (5. 10 W = 0.5Jx20Hz, 6. 10 W = 2Jx5Hz, 7. 60 W = 1Jx60Hz, 8. 60 W = 2Jx30Hz)

**Row 2**: Moses Distance: Soft Stone (9. 10 W = 0.5Jx20Hz, 10: 10 W = 2Jx5Hz, 11. 60 W = 1Jx60Hz, 12: 60 W = 2Jx30Hz); Hard stone (13. 10 W = 0.5Jx20Hz, 14. 10 W = 2Jx5Hz, 15. 60 W = 1Jx60Hz, 16. 60 W = 2Jx30Hz)

**Row 3**: Standard Short Pulse: Soft stone (17. 10 W = 0.5Jx20Hz, 18. 10 W = 2Jx5Hz, 19. 60 W = 1Jx60Hz, 20. 60 W = 2Jx30Hz); Hard Stone (21. 10 W = 0.5Jx20Hz, 22. 10 W = 2Jx5Hz, 23. 60 W = 1Jx60Hz, 24. 60 W = 2Jx30Hz)

**Row 4**: Standard Long Pulse: Soft Stone (25. 10 W = 0.5Jx20Hz, 26. 10 W = 2Jx5Hz, 27. 60 W = 1Jx60Hz, 28. 60 W = 2Jx30Hz); Hard Stone (29. 10 W = 0.5Jx20Hz, 30. 10 W = 2Jx5Hz, 31. 60 W = 1Jx60Hz, 32. 60 W = 2Jx30Hz)

## Discussion

In the present study, preclinical data regarding AR using the Moses Technology^Ⓡ^ in Contact and Distance Mode were compared to those of conventional short- and long-pulse modes of the Ho:YAG laser in artificial hard and soft stones and in different energy and frequency settings using LP and HP lithotripsy. Although the use of real stones could extract more interesting results, the utilization of artificial stones provides the ability to compare and assess the results of different modalities and power settings in the same stone type. Moses Technology^Ⓡ^ consists of a dual-phase single pulse. A vapor bubble is formed by a controlled amount of energy and, consequently, this bubble separates the water. The phenomenon is known as the ‘Moses effect’. The remnant energy is then delivered to the target stone through the vapor bubble [1,11,12,17]. Nonetheless, official high-speed videos show that the effect consists of two laser pulses with a short time interval between them rather than a single ‘modulated’ laser pulse [11]. Data from surgical experts´ opinions [11,12] as well as in vitro [3,7,8,16] and in vivo experiments [10] have been published regarding its efficacy. Mullerad *et al*. described reduced operating time and higher AR, although no statistical significance was observed [18]. In vitro experiments revealed reduced stone retropulsion with Moses Technology^Ⓡ^ [3,7,8,16] leading to higher stone AR compared to conventional pulse modalities [7,10]. In addition, data from in vivo experiments in porcine kidneys depicted that Moses modes are associated with lower stone retropulsion [10].

The first preclinical data were published by Elhilali and colleagues in 2017 [10]. Calculi ablation volumes were 160% greater when the Moses modality was activated probably because stone retropulsion was reduced 50-fold, especially in dusting mode [10]. Regarding the fiber tip to stone distance, Ibrahim *et al*. found that fragmentation efficiency was higher in Moses contact mode. Besides, a significant reduction in stone retropulsion and consequently in operating time was recorded [7]. In contrast, Aldoukhi and colleagues [8] and Winship *et al* [16]. published that efficiency was higher when the fiber tip was 1 mm away from the stone. More specifically, Aldoukhi*et al*. evaluated the effect of fiber tip to stone surface distance on fragmentation with a 3D positioning system, a 230 μm core laser fiber and confocal microscopy. They tested the ablation volume positioning the fiber tip at 0, 0.5, 1, 2, and 3 mm from the stone. The energy and frequency settings used were 1Jx10Hz over 3 min in all tests. The greatest ablation of all pulse modalities was recorded at Moses Distance with a laser fiber tip to stone distance of 1 mm [8]. Our results revealed that in soft stones, the Moses Contact and Moses Distance modes are superior in the dusting (high-frequency, low-energy) and fragmentation (low-frequency, high-energy) settings, respectively. In hard stones, Moses Contact does not seem superior in any group, while Moses Distance seems to be associated with high AR in LP dusting and HP fragmentation settings.

In the current study, four different energy and frequency settings were tested with two different power settings. The Moses Distance mode worked better with 2 J in both HP and LP. This is probably due to the higher energy required for the second bubble to reach its target. This phenomenon was more pronounced in HP lithotripsy. We additionally showed that Moses Distance was more effective compared to Moses Contact in stone AR. These findings are in accordance with those of Aldoukhi and colleagues [8] and Winship [16] and opposite to Ibrahim *et al*. [7]. Further investigations regarding the clinical advantages of one mode compared to the other are deemed necessary. In general, Moses modes showed better outcomes in terms of AR in comparison with non-Moses in soft stones. The clinical implications of this might lay in cases where there is no risk of retropulsion like stones fixed in a calyx. However, when convenient energies (2 J in LP and 1 J in HP) were used, better outcomes were observed with long-pulse mode, and Moses did not seem to improve hard stone AR. In other words, specific settings did not require Moses-On setting, since better results were achieved with long-pulse modulation. Overall, when dusting settings were used, a deeper single cavitation was observed rather than small holes. This could be explained by the fact that dusting leads to less retropulsion and restricted stone movement resulting in the laser beam shooting at a single location of the stone. Likewise, the long-pulse modality and Moses distance resulted in more small fragments compared to short pulse and Moses Contact, overall. This could be explained by the fact that the laser shoot with Moses Contact and Short Pulse was more direct and targeted than long pulse and Moses distance.

To our knowledge, this is the first study to assess AR comparing Moses Contact, Moses Distance, long and short conventional pulses using various energy and frequency settings in hard and soft artificial stones. Previous in vitro studies were based on models mimicking unobstructed stones where the reduction of retropulsion enhanced stone ablation and shortened the procedure time.

### Study limitations

The present study shows certain limitations that must be addressed. Firstly, two stone types (hard and soft) as well as four energy and frequency settings were only used. Nevertheless, these settings are the most widely used, thus, the conclusions may influence the clinical practice. Furthermore, despite the conduction of 96 measurements, the variety of stone-power-pulse combinations resulted in only three stones per combination. Therefore, only descriptive statistics could have been used throughout the study. Additionally, certain technical and clinical issues such as active irritation, temperature rise, etc., should further be studied and analyzed. In the experiments, gravity irrigation instead of active irrigation was used. Moreover, the temperature rise was not evaluated as it has been evaluated in previous studies conducted by our team [19,20]. In addition, fiber damage, burnback of laser fiber and light flashes that occur during HP lithotripsy were not evaluated. The present descriptive in vitro study aimed to compare Moses Technology^Ⓡ^ to conventional modes of holmium laser in soft and hard stones under HP and LP lithotripsy settings and provides useful data regarding what trends can be expected in terms of ablation efficacy using different pulse modulation modalities in Ho:YAG lithotripsy.

## Conclusions

The most effective pulse modality in terms of stone AR depends on stone hardness as well as the energy and frequency settings used. In both hard and soft stones, ablation is more effective when HP (60 W), high-energy (2 J) and Moses Distance are utilized. Further quantitative preclinical and clinical data are deemed necessary to confirm our findings.

## Data Availability

Data available on request from the corresponding author.
